# Selections

**Published:** 1817-12

**Authors:** 


### PART III.

### SELECTIONS.

—— Factorum est copia nobis.

### FOREIGN WORKS.

### I. PHYSIOLOGY.

1. *Prolonged Abstinence.*—The celebrated imposture of the Tutbury fasting woman must yet be fresh in the recollection of our readers. By ourselves, the assertions of Ann Moore, and her accomplices in the iniquitous deception, were never, for a moment, credited. Not only did the system of petty, though obvious artifice, which she hourly practised, appear utterly inconsistent with the open and unsuspicious deportment naturally resulting from consciousness of integrity, but we never could persuade ourselves, that the external signs of health and vigour, which she exhibited, would be long sustained during a perfect suspension of the process of nutrition. The possibility that this process might be well-nigh subverted in the animal œconomy, *when suffering from disease*, without extinction of the vital principle, we did not attempt to deny. There is nothing in the notion at all repugnant to reason or experience. Indeed, the history of medicine presents several facts of this kind, so cautiously observed and recorded, and sustained by such an overwhelming weight of evidence, that no honest and enlightened mind can hesitate to admit them. But what is the state of existence, if existence it can be termed, which the wretched victims of prolonged abstinence have commonly displayed? They can scarcely be said to have lived: they have rather vegetated amid the total destruction of sensation and of thought, of all the attributes and propensities which once distinguished them from the plant, and the death-like torpor of the organs by which they were before connected with the external world. Of this dreary condition, however, the ensuing account* will afford a more striking and expressive portrait than any language of ours can supply. It was transmitted by M. de Varennes, Mayor of Coulomiers, to Professor Chaussier: and, by the latter, communicated to the Society of the Faculty of Medicine of Paris. We see no reason to doubt its authenticity.

While residing at Aire, in 1783, M. de Varennes heard of a

* *Note sur une fille qui a été près de onze ans sans prendre aucun aliment solide, &c. Gazette de Santé,* Mars 1817.

woman, named Marie-Joseph Dahl, a native of the neighbouring village of Disougin, who, for several years, had taken no solid aliment. Incited by this extraordinary rumour, he repaired to the spot, and found the wretched woman, then aged 41, stretched, or rather squatted, on a bed of straw. The trunk was inclined forward, the limbs much bent, and the head supported on one of the knees. A large and coarse napkin, spread upon the straw, served her for a sheet, and a second for a coverlet: she had no other clothing. The skin was whitish; the emaciation extreme. For ten years, the poor creature was reported to have been in this immovable condition, completely destitute of sensation and consciousness; and to have taken, during the whole time, no nutriment except water slightly sweetened with honey, and there called *petit-lait*. Never had she once, in those ten years, of herself, altered her position, nor exhibited any sign of life, except an almost imperceptible respiration, and an occasional motion of deglutition. In any attempt to separate the arms from the body, or one knee from the other, the same resistance was experienced, as is met with in separating a branch, near its origin, from the trunk of a tree. The permanence of this state was attested by some respectable persons of the place, particularly by the curate, a very intelligent man. M. de Varennes visited the woman three times, at intervals of three days, without remarking any change in her condition; which appears to have been the effect of excessive labour and disappointed love. She had formerly been the servant of a farmer, between whose son and herself an attachment subsisted. The father was averse to the connection; but one day, in harvest-time, pointing to a field of wheat, jocosely told Marie, that if, within three days, she could cut the whole without assistance, he would no longer oppose his son's inclination. The poor girl set instantly to work; laboured night and day; and, at last, fell into the melancholy state which has here been described.

At the period of M. de Varennes' visits, two spoonfuls only of *petit-lait* were given to the woman, night and morning; and part of this was spilt. In the event of its being neglected, she never evinced any sign of want. A slight flushing of the face was remarked in a few minutes after this beverage had been swallowed. At times, a yellowish matter, somewhat less liquid than the aliment, was voided by stool. As the jaws were closely locked, three teeth had been driven out to facilitate the introduction of liquids; and, during this operation, and the repeated employment of blisters and cupping-glasses, poor Marie never exhibited the slightest sensibility. She died in 1784, one year after M. de Varennes' visit, in which he had been accompanied by M. Gilet, surgeon of the regiment, and eleven from the commencement of her afflicting malady.

We regret that the history of this otherwise interesting case is rendered imperfect, by the utter silence of the narrator with respect to the temperature and condition of the skin, and the state of the renal and uterine functions.

2. *Disorder of the external Senses.* A curious instance of "quadruple vision"* has been mentioned lately by a German writer. It occurred in a woman, upwards of 30, after a difficult labour, attended with copious hæmorrhage. Every object appeared four-fold. Her enfeebled condition required a light and nutrient diet; and she took, as a tonic mixture, the extract of willow in cinnamon water. Meanwhile, the eye-brows and temples were rubbed, night and morning, with a solution of cajeput oil in Hoffmann's anodyne (sp. ætheris sulph. comp.); and, by a fortnight's perseverance in this plan, perfect vision was restored.

### II. PATHOLOGY and PRACTICE *(Medical).*

1. *Paralysis.* An authentic "report of the illness and death of Madame de Staël"† cannot fail to be perused with interest in this country. The view of the present state of medical opinion and practice in the French capital which the case exhibits, and the celebrity which the subject of it had attained in the literary and political world, alike conspire to incite curiosity and fix attention. Madame de Staël obviously possessed the means of procuring the best medical advice which France, or even the continent, could furnish; and of this privilege, she appears to have somewhat liberally availed herself. The narrative before us is the production of the well-known veteran, Portal, who had been the lady's physician from her early youth, and watched her disease to its fatal termination.

Portal, it seems, was consulted by Madame de Staël, on her return to Paris, on account of an œdematous swelling of the legs, with which she had been for some time troubled. Her complexion, naturally brown, had assumed a deeper shade, and her eyes a yellow colour. She suffered from indigestion and sleeplessness, for which she had long had recourse to the nightly use of opium. Although in her fifty-third year, she had not long ceased to menstruate. Slightly aperient diuretics were‡ prescribed; which, acting on the urinary organs, speedily diminished the œdema. But Madame de Staël, dreading the recurrence of a diarrhœa, with which she had been some time before affected, and which had given way to tonics, combined with a larger than her ordinary dose of opium,

---

* *Vierfaches Sehen, Journal der Practischen Heilkunde,* April 1816.

† *Notice sur la maladie et la mort de Madame la Baronne de Staël, par M. Portal.* Journal Universel des Sciences Medicales, Avril.—Gazette de Santé, Aout, 1817.

‡ Four pills, composed of soap, extract of gentian, hop, and soap-wort, incorporated with ox-gall, were taken fasting in a morning, with two cups of a solution of nitrate of potash in a ptisan prepared with the roots of squitch-grass and dock, and the leaves of hart's tongue and chervil.

not only discontinued the plan, but consulted another physician. By him, irritating powders were prescribed, which provoked slight vomiting, and suppressed the alvine discharge, determined by the former treatment. The patient now profited of a short interval to mingle in the society of Paris. But, the œdema returning in an aggravated form, and the complexion assuming a yellow colour more deep than ever, Portal was again summoned, and resumed his original plan. This was promptly successful; but the lady, once more alarmed by the operation of the medicine on the bowels, refused to continue it, and had recourse to the opinion of a third physician; who recommended opium in larger doses than she habitually employed. Thus the diarrhœa was speedily arrested, and the quantity of urine greatly diminished: the skin resumed a deeper hue; and such a state of drowsiness was induced as to excite alarm. On being again sent for, Portal "found in the pulse an unequivocal movement of fever." The urine was scanty and very red; and deposited a sediment of still deeper colour. The tongue, lips, and cheeks were red; the rest of the face of a deep yellow, and somewhat swollen. The hands, and particularly the feet, were œdematous. Lemonade, with the addition of nitric ether, and purgative injections, were prescribed. The drowsiness subsided, but fever, with a marked evening exacerbation, was developed; and the urine continued scanty, red, and thick.

The opinion that this disease was bilious fever, appeared to be confirmed by a tense state of the right hypochondrium. Hence the quantity of lemonade was diminished, and chicken broth and other drinks, with an addition of nitre, were substituted for it, and seconded by emollient injections. Towards the seventh day, the use of the waters of Vichy was added to this plan. At first, they were administered in combination with chicken-broth; afterwards pure; and, towards the close of the disease, with an addition of acetate of potash.

Under this treatment the fever daily declined, and terminated about the fourteenth day. The urine had progressively become clear and copious, and the fæces passed from a greyish to a yellow colour. The tumefaction of the face and limbs was also considerably reduced, and the region of the liver less prominent and hard. That of the spleen continued swollen. Called in towards the decline of this attack, M. Lucas, sanctioned Portal's plan; and particularly directed increased doses of acetate of potash to be given in the Vichy water. The stools continued bilious, but not too abundant.

Madame de Staël seemed to amend, and was advised to quit her bed; but, from difficulty in preserving the erect posture, aggravated during progression, she was soon obliged to return to it. Pains and spasms of the lower limbs were now the subject of complaint. The urine decreased in quantity, and the skin resumed its yellowish tint; there were also borborygmi, and elevation of the abdomen without tension. The œdema of the extremities reappeared in a very considerable degree. By the employment of diuretics and purgative injections, the quantity of urine again increased. Yet the œdema, or rather anasarca, which supervened, was thought to indicate the application of blisters to the legs, more especially as the patient had, for several years, suffered from an herpetic eruption on the face, now no longer existing. The Vichy water was exchanged for the juices of dandelion, borage, and other plants, with oxymel of squills, nitric ether, digitalis, bruised millepedes, and infusion of hop; and frictions, with powder of digitalis, in a mucilaginous liquid, were also prescribed. By these means, Madame Staël recovered so far as to go out repeatedly in her carriage; but the change was not permanent. She one day complained that, in mounting this vehicle, she had bruised her leg; and that in consequence of great numbness and debility in both the superior and inferior extremities, especially the latter, she was absolutely unable to walk or stand. Nevertheless, her limbs were, at intervals, very painful.

Another physician, now consulted, regarding the liver as the seat of the disease, recommended mercury: but to this prescription Portal could not assent, for he considered that the symptoms originated not solely from the state of the liver, and hence that the mercurial treatment was contra-indicated, more especially as the mouth exhibited an appearance of aphthæ. This plan, nevertheless, was commenced; but, from the farther progress of the disease, it was speedily laid aside.

An eminent accoucheur, who had been sent for to examine the condition of the uterus, thought that he discovered an increase of volume in the organ; but, by an able surgeon, it was declared to be perfectly sound. The latter believed that the spinal marrow was diseased, and that there was an effusion into the vertebral canal. He proposed the application of blisters along the spine, and trial of a stimulating liniment upon the back and extremities. The latter only was adopted. In numerous other consultations, remedies nearly similar were recommended, without success. Frictions, with tincture of phosphorus, availed nothing. The difficulty of moving the lower limbs increased, while the superior, and especially the fingers, had acquired somewhat greater mobility. Yet the patient complained of a sense of constriction in the higher part of the chest; whereon a physician, recently consulted, directed a large blister to be applied.—Another well-known practitioner imagined that he had discovered incipient dropsy of the chest; and that, by means of a funnel of paper, with its base resting on the thorax, and the point introduced into his ear, he could detect a sort of undulation in this cavity. Such a method of examining the interior of the breast was deemed alike extraordinary and inconclusive by Portal; who objected to the inference drawn from it, by remarking, that the urine was now freely evacuated; all the dropsical symptoms had well nigh disappeared, and the patient could lie in the horizontal posture for a whole day. The same physician directed against the prevailing spasms the application of two magnetic plates to the thorax. The insufficiency of this remedy was soon apparent.

Meanwhile, Madame de Staël continued to waste, and experience numbness in the limbs, with total impracticability of progression. Asses' milk was therefore recommended. The pulse was often contracted, small, and frequent, with coldness of the skin and extremities: subsequently, it rose, became more soft and developed, with an increase of temperature. Stimulant and sedative remedies were alternately employed as the pains varied.

A celebrated surgeon, of Geneva, summoned to a consultation with the Paris physicians, proposed the internal use of mustard, and the employment of stimulating ointments on the vertebral column. This plan, in combination with infusion of cinchona, which, on account of increasing signs of debility, had been previously prescribed, was continued for some days; but, notwithstanding, there supervened a retention of urine, which could only be obviated by the assistance of the catheter.

But gangrene had already commenced in the region of the coccyx, and there were some spots of bad character on the left lower limb. To the rapid progress of this affection, cinchona and every other remedy were vainly opposed. The fatal event took place at four o'clock in the morning of July the 14th. Madame de Staël preserved the energy of her intellect to the last. She passed nearly the whole day preceding her death, seated on an arm-chair, in conversation with her friends. She retired to bed at the usual hour; slept awhile; and, on awaking, requested that her nightly dose of opium might be administered. This taken, she again fell asleep, and shortly afterwards she was found dead.

The body was opened in order to be embalmed, and conveyed to Coppet, in Switzerland, the birth-place of Madame de Staël. Portal was not invited to the operation; but he was informed by M. Jurine, who witnessed it, that neither hydrothorax, nor alteration of the brain and spinal marrow, nor effusion into the vertebral canal was discovered; that the other viscera were sound, and the liver only indurated, and somewhat tumefied in some portions of its extent. Portal, however, thinks that the sole cause of such a formidable disease could not consist in this slight alteration. He attributes it rather to a cachexy, or bad disposition of the body, which may proceed from various antecedent causes, rendered still more intense by the bilious fever. Hence resulted a morbid affection of the nerves of the abdominal plexus, which are distributed to the liver and other biliary organs—and of the spinal marrow and nerves sent off from it to the trunk and extremities. Thus, he contends, were produced the paralysis of the latter, the thoracic pains, and lastly, the fatal gangrene which supervened.

It has repeatedly been our lot to observe incurable paralysis and gangrene ushered in by the most strongly-marked symptoms of hepatic congestion and disorder. But we never yet have had an opportunity of ascertaining the seat and character of the internal morbid appearances essentially connected with this form of disease. Boerhaave, Wepfer, and Morgagni, as Portal remarks, were persuaded that paralysis of the trunk, and especially of the lower limbs, might result, independently of any evident lesion of the brain or spinal marrow, from morbific congestion of the cellular structure, or of the nerves themselves forming the different abdominal plexuses that communicate with the vertebral nerves, from which the trunk and extremities are supplied.* This appears to us to be sound pathological doctrine. We have, sometimes, seen mercury and counter irritants, when early and judiciously employed, productive of benefit in this obscure and commonly fatal affection.

---

### PART IV.

### MEDICAL MISCELLANIES.

Quicquid agunt Homines.

*On the Laws of Muscular Motion.* By J. R. Park, M. B. &c.

* *Morgagni, de sed. et caus. morb.* Ep. ii. Art. 20.—*Portal, Anat. Med.* tom. iv. p. 143.

